# Walnut Consumption for Two Years and Leukocyte Telomere Attrition in Mediterranean Elders: Results of a Randomized Controlled Trial

**DOI:** 10.3390/nu10121907

**Published:** 2018-12-04

**Authors:** Tania-Marisa Freitas-Simoes, Montserrat Cofán, Maria A. Blasco, Nora Soberón, Miguel Foronda, Mercè Serra-Mir, Irene Roth, Cinta Valls-Pedret, Mónica Doménech, Elena Ponferrada-Ariza, Carlos Calvo, Sujatha Rajaram, Joan Sabaté, Emilio Ros, Aleix Sala-Vila

**Affiliations:** 1 Lipid Clinic, Department of Endocrinology and Nutrition, Hospital Clínic de Barcelona, IDIBAPS, Villarroel 170, Edifici Helios, despatx 8, 08036 Barcelona, Spain; FREITA@clinic.cat (T.-M.F.-S.); Mcofan@clinic.cat (M.C.); serramir@clinic.cat (M.S.-M.); roth@clinic.cat (I.R.); cvalls1@clinic.cat (C.V.-P.); mdomen@clinic.cat (M.D.); eponferr@clinic.cat (E.-P.A.); ccalvoanibarro@gmail.com (C.C.); eros@clinic.cat (E.R.); 2 Ciber Fisiopatología de la Obesidad y Nutrición (CIBEROBN), Instituto de Salud Carlos III (ISCIII), 28029 Madrid, Spain; 3 Telomeres and Telomerase Group, Molecular Oncology Program, Spanish National Cancer Research Centre (CNIO), 28029 Madrid, Spain; mblasco@cnio.es (M.A.B.); norasoberon@gmail.com (N.S.); miguel.foronda.alvaro@gmail.com (M.F.); 4 Department of Nutrition, School of Public Health, Loma Linda University, Loma Linda, CA 92350, USA; srajaram@llu.edu (S.R.); jsabate@llu.edu (J.S.)

**Keywords:** aging, alpha-linolenic acid, biomarker, *n*-3 PUFA, nuts

## Abstract

Randomized controlled trials on diet and shortening of leukocyte telomere length (LTL) mostly focus on marine-derived *n*-3 polyunsaturated fatty acids (PUFA). Walnuts are a sustainable source of *n*-3 PUFA. We investigated whether inclusion of walnuts (15% of energy) in the diet for 2 years would maintain LTL in cognitively healthy elders (63–79 years old) compared to a control group (habitual diet, abstaining from walnuts). This opportunistic sub-study was conducted within the Walnuts and Healthy Aging study, a dual-centre (Barcelona, Spain and Loma Linda University, California) parallel trial. A sub-set of the Barcelona site participants were randomly assigned to the walnut (*n* = 80) or control group (*n* = 69). We assessed LTL at baseline and at 2 years and we conducted repeated-measures ANCOVA with 2 factors: time (baseline, 2 years) and group (control, walnut) and their interaction. Adjusted means (95% confidence interval) of LTL (in kb) in controls were 7.360 (7.084,7.636) at baseline and 7.061 (6.835,7.288) after 2 years; corresponding values in the walnut group were 7.064 (6.807,7.320) and 7.074 (6.864,7.284). The time × intervention interaction was nearly significant (*p* = 0.079), suggestive of a trend of walnut consumption in preserving LTL. This exploratory research finding should be confirmed in trials with adequate statistical power.

## 1. Introduction

Population aging is a global trend [1]. Given the socioeconomic burden of treating age-related diseases [2], identifying simple strategies to promote healthy aging (preserved physical and cognitive function, quality of life and independence) have emerged as a major public health concern [3]. In parallel, there is a need for valid biomarkers to aid in better characterizing the aging process. The nine tentative hallmarks of aging listed in 2013 [4] prompted many potential aging biomarkers. To date, the perfect aging biomarker is yet to be discovered. Telomere attrition is one of the suggested hallmarks of aging [4]. In brief, telomeres are repeating DNA sequences (5′-TTAGGG-3′) located at chromosomal ends. They allow cells to distinguish chromosome ends from double-strand breaks and thus protect chromosomes from end-to-end fusion, recombination and degradation. During each cell division, a part of telomere is lost. Telomerase can repair critically short telomeres but its capacity is limited. The critical accumulation of uncapped (short) telomeres triggers cellular senescence, apoptosis and/or the permanent cell cycle arrest in many body tissues. This fostered the use of shortening of leukocyte telomere length (LTL) to help explore the link between diet and aging, despite having inherent caveats [5].

Evidence has accumulated that LTL is susceptible to modification by lifestyle, particularly diet and whether specific nutrients, foods, or dietary patterns can modulate LTL is a topic of increasing interest [6]. An inverse association was observed between the baseline red blood cell (RBC) content of fish-derived *n*-3 PUFA (an objective marker of intake) and the rate of telomere attrition over a 5-year period in patients with coronary artery disease [7]. This seminal finding prompted several randomized controlled trials (RCTs) aimed at testing the effect of consumption of gram doses of marine *n*-3 PUFA on LTL in subjects with overweight [8], mild cognitive impairment [9], chronic kidney disease [10] and women at mid-pregnancy [11]. These studies were always conducted for short periods (≤ 6 months) and none reported significant effects of the dietary intervention on LTL.

In contrast to *n*-3 PUFA from marine origin, the effect of terrestrial (and more sustainable) sources of *n*-3 PUFA on LTL remains unexplored. Walnuts are the commonly eaten food containing the highest amount of alpha-linolenic acid (C18:3n-3, ALA, ≈10 g per 100 g) [12]. In addition, they have a rich matrix of antioxidants, namely polyphenols and vitamin E, which intake has also been related to maintained LTL [6]. Therefore, given the richness of walnuts in bioactive components that have the potential to beneficially impact LTL through additive effects, we hypothesized that inclusion of this food in the regular diet for a long period (2 years) would protect from leukocyte telomere attrition in older subjects.

## 2. Methods

### 2.1. Study Population

This opportunistic sub-study was conducted within the frame of the Walnuts and Healthy Aging (WAHA) study (https://clinicaltrials.gov/ct2/show/NCT01634841), a dual centre (Hospital Clinic, Barcelona, Spain; and Loma Linda University, Loma Linda, CA, USA) RCT conducted in free living, cognitively healthy elderly men and women (63 to 79 years) aimed at examining whether supplementation with walnuts (15% of energy, 30 to 60 g per day, based on estimated energy requirements) can prevent age-related diseases compared to a control diet (usual diet with abstention from walnuts). Exclusion criteria were: morbid obesity (BMI ≥ 40 kg/m^2^), uncontrolled diabetes (HbA1c > 8%), uncontrolled hypertension (on-treatment blood pressure ≥ 150/100 mm Hg), prior stroke or major head trauma, any relevant psychiatric illness, advanced cognitive deterioration (mild cognitive impairment or frank dementia), other neurodegenerative disorders (i.e., Parkinson’s disease), any chronic illness expected to shorten survival, bereavement, allergy to walnuts and customary use of fish oil, flaxseed oil or soy lecithin supplements. The protocol, which has been described in detail [13], was conducted according to the guidelines of the Declaration of Helsinki and was approved by the ethics committee of each institution. Written informed consent was obtained from all subjects. LTL was only assessed in Barcelona site, as described [13].

### 2.2. Assessment of Risk Factors

Hypertension was defined as systolic blood pressure of 140 mm Hg or higher, diastolic blood pressure of 90 mm Hg or higher, or the use of antihypertensive medication. Diabetes was defined as fasting blood glucose level of 126 mg/dL or higher on two occasions, HbA1c ≥ 6.5%, or use of antidiabetic drugs. Dyslipidaemia was defined as a low-density lipoprotein cholesterol level higher than 130 mg/dL, a high-density lipoprotein cholesterol level of 40 mg/dL or lower in men or 50 mg/dL or lower in women or use of lipid-lowering drugs. Smoking status was categorized into never, current or past smoking according to self-reports and pack-years of smoking were calculated. Anthropometric data were obtained as follows: body weight was measured in light clothing and without shoes to the nearest 0.1 kg by calibrated scales. Height was measured to the nearest half centimetre using a wall-mounted stadiometer. BMI was calculated as weight in kilograms divided by height in meters squared. Waist circumference was measured to the nearest 0.5 cm by using an anthropometric tape midway between the lowest rib and at the iliac crest at minimal respiration.

### 2.3. Intervention

All participants followed their habitual diet and the intervention was eating walnuts daily for 2 years or abstaining from walnuts. Participants were randomized to either control or walnut group using a web-based computerized random number table with stratification by centre, gender and age category. Couples entering the study were treated as one number and were randomized into the same group. Participants assigned to the walnut group received ≈15% of their daily energy intake as walnuts. To estimate the required amount of walnuts, at the second visit participants completed a 3-day diet food record. The same tool was also used to monitor dietary intake during the study on a 6-month basis. The nutrient composition of the diets was calculated with Food Processor Plus software (ESHA Research, Salem, OR, USA) adapted to nutrient databases of local foods when appropriate. At baseline and yearly thereafter participants also completed a validated short version of the Minnesota questionnaire [14]. Physical activity was expressed in minutes at a given metabolic equivalent (MET-min) per week. The physical activity factor and the energy requirements were obtained using the WHO formula for energy needs for adults >60 years [15]. The amount of walnuts thus calculated ranged from 30 to 60 g/day. Sachets for daily consumption containing 30, 45 or 60 g of pieced walnuts were provided as 8-week allotments to the participants in the walnut group at the time of their 2-monthly clinic visits with the study dietitian. The participants were given instructions to eat walnuts daily, preferably raw, either as a snack or by incorporating them into recipes and to finish their daily dose either in one sitting or spread them throughout the day. Those who had problems chewing were provided with grinders at no cost and instructed on how to eat the ground walnuts incorporated into semifluid foods such as yogurt. Participants in the control group were instructed to abstain from walnuts and avoid other nuts at doses >2 serving/week for the duration of the study. Once randomized, all participants were scheduled for 2-month visits with the study dietitians aimed at assessing compliance, increasing retention and collecting data on diet adherence, tolerance, medication changes and anthropometry.

### 2.4. Biochemical Analyses

At baseline and at the end of intervention, blood samples were drawn after an overnight fast. Serum lipid and glucose concentrations were determined by standard enzymatic methods in the hospital clinical laboratory. Fresh peripheral blood mononuclear cells were separated from 5 mL of EDTA-collected blood by Ficoll gradient centrifugation (Histopaque^®^-1077, Sigma-Aldrich, St Louis, MO, USA). The resulting pellet of peripheral blood mononuclear cells was re-suspended in Foetal bovine serum (Sigma-Aldrich, St Louis, MO, USA) supplemented with 10% dimethylsulphoxide and stored at −80 °C. An aliquot of whole blood was stored at −80 °C until fatty acids analysis.

Telomere length quantification was carried out by high-throughput quantitative fluorescence in situ hybridization with automated fluorescence microscopy, as described [16]. Briefly, cells were counted and plated (80,000 to 100,000 cells/well) in clear-bottomed black-walled, 96-well plates. The 4′,6-diamidino phenylindole channel was used for nuclear staining and the Cy3 channel was used for telomere detection. LTL was determined using individual telomere spots (N90000 telomere spots per sample). Fluorescence intensities were converted into kb by using L5178-R, L5178-S and CEM cells, which have respective stable telomere lengths of 79.7 kb, 10.2 kb and 7.5 kb as calibration standards [17]. Samples were analysed in duplicate or in triplicate in the case of calibration standards. Mean LTL and % LTL < 3 kb were calculated for each sample. Previous studies have used < 3 kb as the cut-off for “short telomeres” in humans [18]. The intra-assay coefficient of variation was 6.3% for LTL and 10.5% for % LTL < 3 kb, while the inter-assay coefficient of variation was 5.6% for LTL and 12.8% for % LTL < 3 kb.

To objectively determine adherence to supplemental walnuts, we measured the RBC proportions of ALA at baseline and at the end of the intervention, as described [19]. Briefly, a 100-μL aliquot of EDTA-collected blood was haemolyzed, cells were spun and the supernatant was discarded. The pellet (>99.5% RBC membranes) was dissolved in 1 mL boron trifluoride-methanol solution and transferred into screw-cap test tubes, which were heated for 10 min at 100 °C. The fatty acid methyl esters were recovered in n-hexane and separated by gas chromatography using an Agilent 7890A Gas Chromatograph (Agilent España, Madrid, Spain) equipped with a 30 m × 0.25 μm × 0.25 mm SupraWAX-280 capillary column (Teknokroma, Barcelona, Spain), an autosampler and a flame ionization detector. ALA is expressed as percentage of the total identified fatty acids in the sample. We also used gas-chromatography to determine the fatty acid profile of walnuts used in our study. Mean (standard deviation) values (in % of total fatty acids) were 5.3 (0.1) for C16:0; 1.2 (0.1) for C18:0; 27.8 (0.3) for C18:1n-9; 55.6 (0.6) for C18:2n-6 and 9.9 (0.1) for ALA (*n* = 4 samples from different batches).

### 2.5. Statistical Analyses

From April 2012 to December 2013, 642 potential candidates were pre-screened in the Barcelona site by completing short questionnaires and 198 were excluded for not meeting eligibility criteria. Of the remaining 444 candidates formally assessed for eligibility, 92 were excluded after a clinical visit and physical examination. Baseline LTL data were missing for 6 participants. This study was initially conceived as an opportunistic WAHA sub study to be conducted in a subset of participants undergoing sequential brain magnetic resonance imaging (*n* = 120) [13]. However, in order to increase the statistical power, we randomly selected 49 additional participants. This resulted in 169 recruited subjects randomly assigned to one of the two interventions. The flow chart depicting the study and reasons for exclusion can be found in Supplementary Figure S1.

Normal distribution of data was assessed using graphical methods and the Shapiro–Wilk test. Data are expressed as mean and standard deviation (SD) for quantitative variables following a normal distribution. Skewed variables are reported as medians and interquartile ranges, while categorical data are expressed as frequencies and percentages. The effect of the intervention on LTL and % LTL< 3 kb was explored by using repeated-measures ANCOVA with 2 factors: time (baseline vs. 2 y) as repeated measure, group (control vs. walnut) and their interactions, with age, gender and smoking in pack-years as covariates. We also searched for intra-group differences between baseline and final LTL by using repeated-measures ANCOVA with age, gender and smoking in pack-years as covariates. We assessed between-group differences in nutrient intake by ANOVA and the effect of the intervention on the RBC proportion of ALA by using repeated-measures ANOVA with time and group as factors and their interaction. Alternatively, we replaced ALA with the *n*-6 PUFA:*n*-3 PUFA ratio in RBCs. Pearson’s correlation coefficients were used to study the association between 2-y changes in the *n*-6 PUFA:*n*-3 PUFA ratio and 2-y changes in LTL. We assessed between-group differences in incidence of cardiovascular disease, cancer, cataract surgery and trauma admission by Chi-squared test. Statistical significance was set at the *p* < 0.05 level. Analyses were performed using SPSS software, release 19.0 (IBM Corp., Armonk, NY, USA).

## 3. Results

Of the total 169 subjects randomized to the two diets, 162 completed the trial. There were 2 dropouts due to severe dyspepsia attributed to walnuts, while 6 participants had milder dyspepsia that was solved by reducing walnut doses. Additionally, 13 participants were excluded from analyses because of lack of (*n* = 4) or technically unacceptable (*n* = 9) leukocyte specimens. Therefore, complete LTL, dietary, anthropometric and RBC fatty acid data were available for 149 participants (80 walnuts and 69 controls) and subsequent data refer only to them.

**Figure 1:**
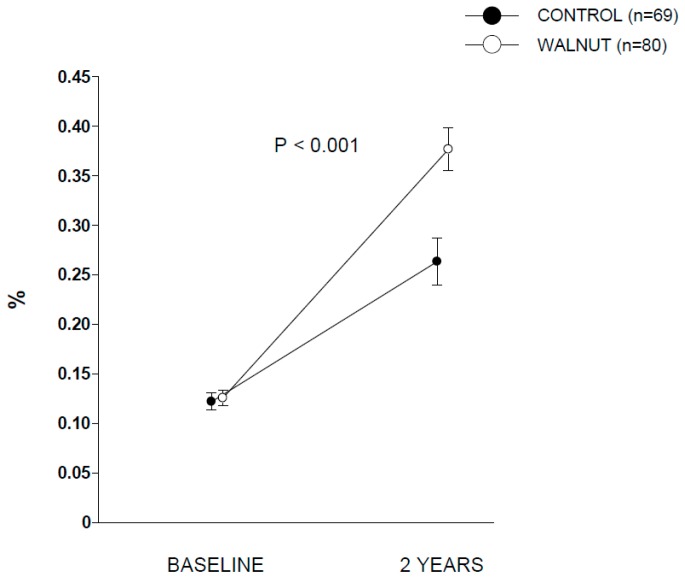
Changes in alpha-linolenic acid (expressed as percentage of total fatty acids) in red blood cells after 2 years of intervention. *p* refers to interaction between time (baseline vs. 2 years) and intervention (control vs. walnut), obtained by two-way repeated measures ANCOVA. Baseline means (95% confidence interval) are 0.122 (0.113 to 0.131) and 0.126 (0.118 to 0.134) for control and walnut group, respectively. Means (95% confidence interval) at the end of intervention were 0.263 (0.240 to 0.287) and 0.377 (0.355 to 0.399) for control and walnut group, respectively.

**Figure 2:**
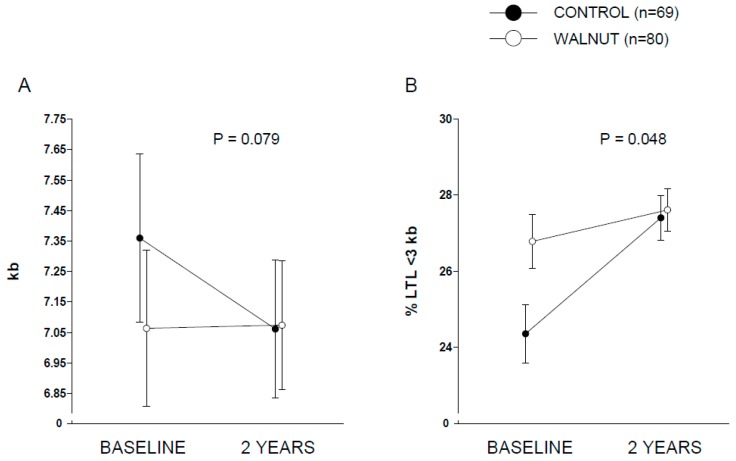
Changes in leukocyte telomere length (panel (**A**), expressed as kb) and in percentage of telomeres with a length <3 kb (panel **B**) in study participants after 2 years of intervention. *p* refers to interaction between time (baseline vs. 2 years) and intervention (control vs. walnut), obtained by two-way repeated measures ANCOVA with age, gender and smoking in pack-years as covariates. For Panel A, adjusted baseline means (95% confidence interval) were 7.360 (7.084 to 7.636) and 7.064 (6.807 to 7.320) for control and walnut group, respectively. Adjusted means (95% confidence interval) at the end of intervention were 7.061 (6.835 to 7.288) and 7.074 (6.864 to 7.284) for control and walnut group, respectively. For Panel B, adjusted baseline means (95% confidence interval) were 24.36 (22.84 to 25.88) and 26.78 (25.38 to 28.19) for control and walnut group, respectively. Adjusted means (95% confidence interval) at the end of intervention were 27.40 (26.23 to 28.57) and 27.61 (26.52 to 28.70) for control and walnut group, respectively.

Table 1 displays baseline clinical characteristics. Baseline nutrient content of the self-reported diets are reported in Table 2. At the end of the trial, participants in the walnut diet increased dietary energy and total fat and reciprocally decreased carbohydrate compared to the control diet. Reflecting the nutrient composition of walnuts, walnut diet participants also increased intake of linoleic acid and ALA (Table 2). Changes in ALA intake expressed as g/day were 3.55 (95% confidence interval (CI), 3.37 to 3.73) and −0.02 (95% CI, −0.22 to 0.17) for the walnut and control groups, respectively (*p* between groups < 0.001). Compliance with walnut ingestion was >98% according to both participants’ reports and recount of empty packages. The analysis of RBC ALA during the two periods disclosed no between-group differences at baseline. At the end of the intervention, participants in walnut group showed significant increases in RBC proportion of ALA compared to control group (Figure 1), confirming good adherence to the intervention. Supplementary Table S1 contains additional data for RBC proportions of other selected fatty acids. Figure 2 displays covariate-adjusted changes in LTL (panel A) and % LTL < 3 kb (panel B). A nearly significant (*p* = 0.079) time × intervention interaction was observed for LTL, while full albeit marginal statistical significance was observed for % LTL < 3 kb (*p* = 0.048). Raw changes in LTL from baseline in both groups are shown in Supplementary Figure S2. Changes in LTL (in kb) were 0.01 (95% CI, −0.22 to 0.24) and −0.29 (95% CI, −0.56 to −0.03) for the walnut and control groups, respectively. Values for % LTL < 3 kb were 0.88 (95% CI, −0.58 to 2.33) and 2.98 (1.34 to 4.63) for the walnut and control groups, respectively. This overall suggests an effect of walnuts in preventing leukocyte telomere attrition compared to control participants. No significant differences were found between the two groups regarding changes in the *n*-6 PUFA:*n*-3 PUFA ratio in RBCs (*p* for interaction time × intervention = 0.281). The Pearson’s correlation coefficient between 2-year changes of the *n*-6 PUFA:*n*-3 PUFA ratio in RBCs and 2-year changes in LTL was 0.113 (*p* = 0.175). No significant between-group differences were observed regarding incident cardiovascular disease (walnuts, *n* = 1; control, *n* = 2), cancer (walnuts, *n* = 3, with one case diagnosed 1 month after entry into the trial; control, *n* = 1), trauma admission (walnuts, *n* = 5; control, *n* = 5) and cataract surgery (walnuts, *n* = 3; control, *n* = 4).

## 4. Discussion

In this opportunistic sub-study within a larger RCT conducted in cognitively healthy elders, we investigated whether intervention with a diet supplemented with walnuts at 15% of daily energy (~30 to 60 g/day) for 2 years would delay telomere shortening compared to a control diet (abstaining from walnuts). We found a nearly significant (*p* = 0.079) interaction between time (baseline vs. 2 years) and dietary intervention (control vs. walnuts), suggestive of potential effect of walnut consumption in preventing telomere attrition. However, the relatively short period of intervention and the sample size precluded detection of the effect of intervention on incident age-related diseases.

Promoting healthy aging is of utmost public health importance. There is increasing interest in understanding the mechanisms underlying the process of aging. Attrition of telomeres, structures located at the chromosomal end, leads to senescence and destruction of some cells. This prompted the measurement of telomere length of circulating cells as a biomarker of aging. It has been suggested that telomeres are highly susceptible to oxidative stress; hence they are plausible targets for antioxidant treatment. Non-observational research on the topic is in its infancy, with RCTs mostly testing marine-derived *n*-3 PUFA in a short intervention (≤6 months) [8,9,10,11]. In the present 2-year RCT, we investigated whether LTL of healthy elders changed after supplementing their habitual diet with walnuts. The optimal composition of walnuts in bioactive micronutrients, including sizable amounts of ALA (the vegetable *n*-3 PUFA) but also melatonin and polyphenols [20], was hypothesized to act synergistically in reducing inflammation and oxidative stress, hence curbing leukocyte telomere attrition.

Our trial had slight differences compared to the RCTs testing marine-derived *n*-3 PUFA on LTL. First, rather than testing a single nutrient with long-known salutary effects (long-chain *n*-3 PUFA) given in isolation (concentrated fish oil capsules), we tested a whole food, making difficult to disentangle the exact contribution of each nutrient and bioactive to changes in LTL. Second, whilst RCTs to date were conducted in overweight subjects aged 40–85 years old (8), elders with mild cognitive impairment [9], or patients with chronic kidney disease [10], our study population was made of cognitively healthy individuals aged ≥ 63 years. However, attrition rate does not differ that much within the age range of these studies [21]. Finally, compared to previous trials, the sample size was larger and the duration of intervention was longer (2 years instead of 6 months). This probably contributes to explain LTL differences observed between our control group (adjusted mean, −290 bp (95% CI, −560 to −30)) and, for instance, the control group of a study involving 4 months of intervention (−43 bp (95% CI, −131 to 46)) [8].

Another aspect should be underlined. One of the most remarkable findings from the RCT conducted by Kiecolt-Glaser and co-workers [8] was that participants who increased the plasma *n*-3 PUFA:*n*-6 PUFA ratio disclosed lower telomere shortening at the end of the intervention. We also tackled this issue but replacing plasma with RBCs, which turnover (120-day lifespan) makes them a better choice for accurately assessing long-term *n*-3 PUFA intake [22]. In the present study, we observed no between-group differences in the *n*-6 PUFA:*n*-3 PUFA ratio. This might be explained by the fact that, in contrast to the Kiecolt-Glaser et al. study [8], the exposure tested in our study (walnuts) not only supplied *n*-3 PUFA but also substantial amounts of the *n*-6 PUFA linoleic acid (C18:2n-6), thus maintaining the ratio essentially unaffected.

Our study has limitations. This is an opportunistic study conducted in a subset of the WAHA participants at the Barcelona site. The parent RCT was not specifically designed to test this hypothesis. The two arms of the study displayed differences regarding baseline LTL. The number of participants is small but still larger than those examined in RCTs of marine-derived n-3PUFA and with longer exposure time (2 years, compared to ≤ 6 months). Our results should be considered exploratory and need to be confirmed. Further RCTs with adequate statistical power are needed to ascertain not only whether LTL can be modified through consumption of specific foods and/or adherence to particular dietary patterns but also how this affects frailty and premature aging. Finally, because the study participants were elders having general good health and living in a Mediterranean country, our findings cannot be easily extrapolated to other populations. Our study also has strengths, such as being conducted in free-living individuals, making it as close to real-life conditions as possible, since there were no major diet changes other than either eating walnuts daily or abstaining from them. In addition, we evaluated adherence to the intervention with an objective biomarker.

In conclusion, we report that inclusion of walnuts in the regular diet for 2 years tends to delay leukocyte telomere attrition in older individuals compared to those following their usual diet and abstaining from walnuts. This suggests that walnuts, a sustainable source of plant-derived n-3PUFA and antioxidants, might impact the aging process. Future research is warranted, in particular to identify individuals who can obtain the greatest benefits from such simple, low-cost dietary intervention and delineating how it can translate into primary prevention against age-related diseases.

## Figures and Tables

**Table 1:** Baseline characteristics of the study participants according to intervention group.

Variable	Walnut Group (*n* = 80)	Control Group (*n* = 69)
Female sex, *n* (%)	57 (71.3)	48 (69.6)
Age, years	68.6 (3.2)	68.7 (3.2)
Family history of premature CVD, *n* (%)	4 (5)	2 (2.9)
Body mass index, kg/m^2^	26.4 (3.6)	27.2 (4.2)
Waist circumference, cm	96.5 (10.7)	99.5 (12)
Physical activity, METs-min per week	2728 (1789 to 4187)	2306 (1512 to 3531)
Ever smoking, *n* (%)	26 (32.5)	21 (30.4)
Smoking, pack-years	0 (0 to 13)	0 (0 to 8)
Hypertension, *n* (%)	40 (50)	42 (60.9)
Type 2 diabetes, *n* (%)	10 (12.5)	7 (10.1)
Treated with metformin, *n* (%)	7 (8.8)	3 (4.3)
Fasting glucose, mg/dL	93 (85.2 to 102)	91 (85.5 to 98.5)
Haemoglobin A1c, %	5.7 (5.5 to 6.1)	5.8 (5.6 to 5.9)
Dyslipidaemia, *n* (%)	46 (57.5)	33 (47.8)
Treated with statins, *n* (%)	33 (41.3)	19 (27.5)
Total cholesterol, mg/dL	207 (39)	211 (32)
HDL cholesterol, mg/dL	59 (16)	61 (14)
LDL cholesterol, mg/dL	128 (34)	131 (26)
Triglycerides, mg/dL	90 (69 to 123)	88 (70 to 114)

Values are means (SD) except for quantitative variables (expressed as *n* and %) and physical activity, smoking in pack-years, fasting glucose, haemoglobin A1c and triglycerides, expressed as medians (interquartile ranges). CVD, cardiovascular disease; METs-min, minutes at a given metabolic equivalent level (units of energy expenditure in physical activity; 1 MET-min is roughly equivalent to 1 kcal); HDL, high-density lipoprotein cholesterol; LDL, low-density lipoprotein cholesterol.

**Table 2:** Baseline energy and nutrient intake and 2-year changes by study group.

Variable		Walnut Group (*n* = 80)	Control Group (*n* = 69)	*p* *
Energy (kcal/d)	Baseline	1742 (1669 to 1815)	1639 (1561 to 1718)	
	Change	129 (55 to 203)	−9 (−88 to 71)	0.014
Total protein (% energy)	Baseline	18.0 (17.3 to 18.7)	18.4 (17.6 to 19.1)	
	Change	−0.6 (−1.4 to 0.2)	0.1 (−0.7 to 1.0)	0.227
Total carbohydrate (% energy)	Baseline	42.1 (40.5 to 43.7)	42.2 (40.4 to 43.9)	
	Change	−3.9 (−5.5 to -2.2)	1.9 (0.1 to 3.7)	<0.001
Total fat (% energy)	Baseline	38.5 (37.2 to 39.8)	39.2 (37.8 to 40.6)	
	Change	6.3 (4.7 to 7.8)	−1.8 (−3.5 to −0.1)	<0.001
Total saturated fat (% energy)	Baseline	9.6 (9.1 to 10.1)	9.8 (9.2 to 10.3)	
	Change	−0.5 (−1.1 to 0.1)	−0.5 (−1.2 to 0.1)	0.946
Total monounsaturated fat (% energy)	Baseline	20.1 (19.2 to 21.1)	20.8 (19.8 to 21.8)	
	Change	−1.5 (−2.7 to −0.4)	−1.3 (−2.5 to −0.1)	0.801
Linoleic acid (% energy)	Baseline	4.2 (3.8 to 4.6)	4.6 (4.1 to 5.0)	
	Change	6.7 (6.2 to 7.3)	−0.6 (−1.2 to 0.0)	<0.001
Alpha-linolenic acid (% energy)	Baseline	0.41 (−0.38 to 0.44)	0.44 (0.41 to 0.47)	
	Change	1.71 (1.62 to 1.79)	−0.01 (−0.10 to 0.08)	<0.001
EPA + DHA (% energy)	Baseline	0.21 (0.16 to 0.26)	0.17 (0.12 to 0.23)	
	Change	−0.03 (−0.09 to 0.03)	0.01 (−0.05 to 0.08)	0.283
Total fibre (g/d)	Baseline	18 (16 to 19)	17 (16 to 19)	
	Change	2.1 (0.8 to 3.4)	0.5 (−0.9 to 1.9)	0.108
Cholesterol (mg/d)	Baseline	255 (233 to 277)	241 (217 to 265)	
	Change	−18 (−44 to 9)	−10 (−38 to 19)	0.678

Data expressed as means (95% confidence interval). EPA denotes eicosapentaenoic acid; DHA, docosahexaenoic acid.* *p* value obtained by ANCOVA adjusted for age and sex. Changes for which the 95% confidence interval does not include zero are significantly different from the baseline.

## Supplementary Materials

The following are available online at http://www.mdpi.com/2072-6643/10/12/1907/s1, Figure S1: Flow-chart of the study, Figure S2: Changes in LTL in all participants from the two groups after 2 years of intervention, Table S1: Baseline and 2 year red blood cell proportions of selected fatty acids by intervention group.
