# Supplementary material for: Walnut Consumption for Two Years and Leukocyte Telomere Attrition in Mediterranean Elders: Results of a Randomized Controlled Trial
Source: Nutrients. 2018 Dec 4;10(12):1907. doi: 10.3390/nu10121907 (PMC6316673; doi:10.3390/nu10121907)
Supplement: Supplementary file 1 [file nutrients-10-01907-s001.pdf]

Supplementary material of **"Walnut consumption for two years and leukocyte telomere attrition in Mediterranean elders: results of a randomized controlled trial"** by Tania-Marisa Freitas-Simoes et al.

eFigure 1. Flow-chart of the study

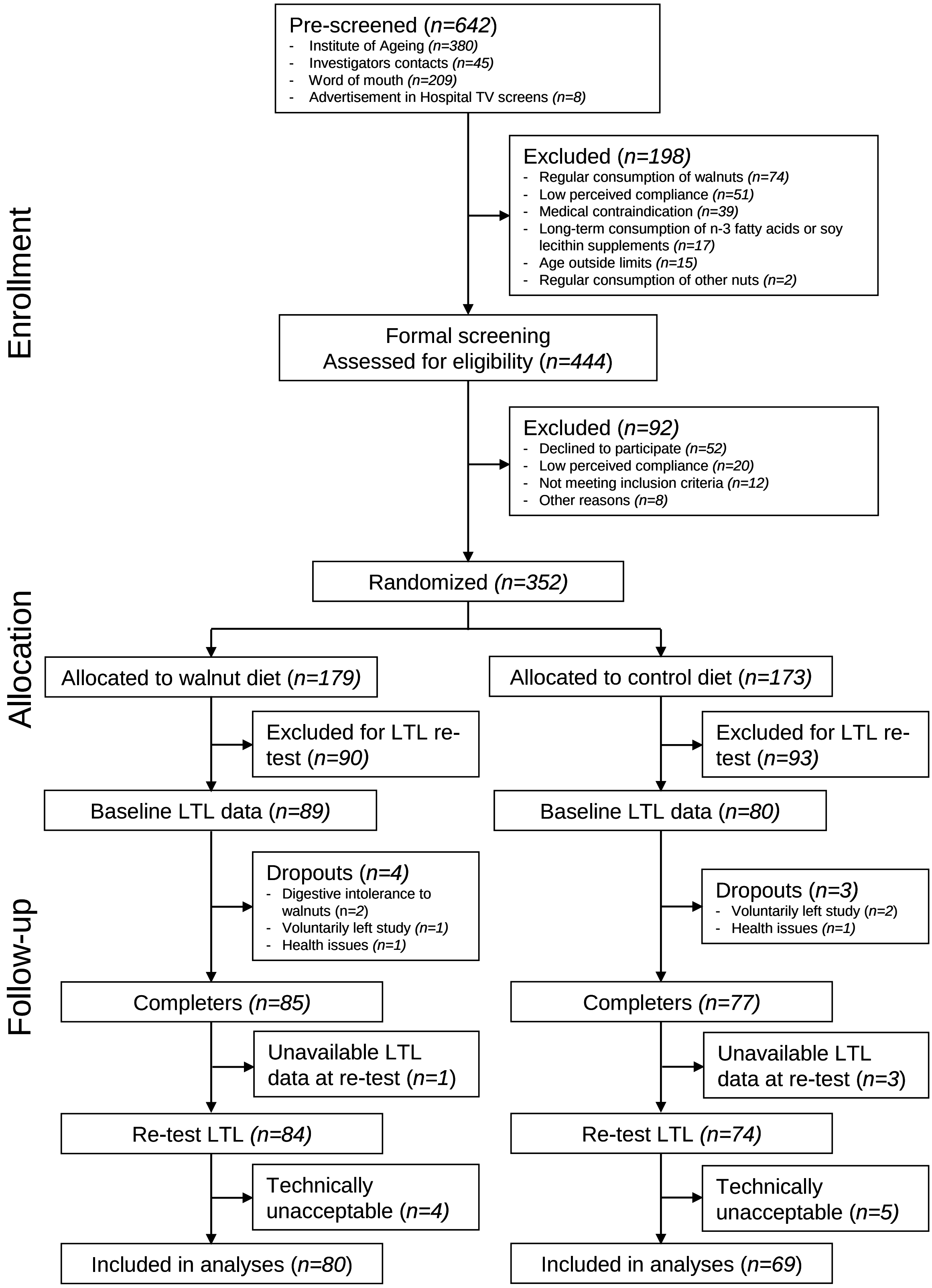

eFigure 2. Changes in LTL in all participants from the two groups after 2 years of intervention.

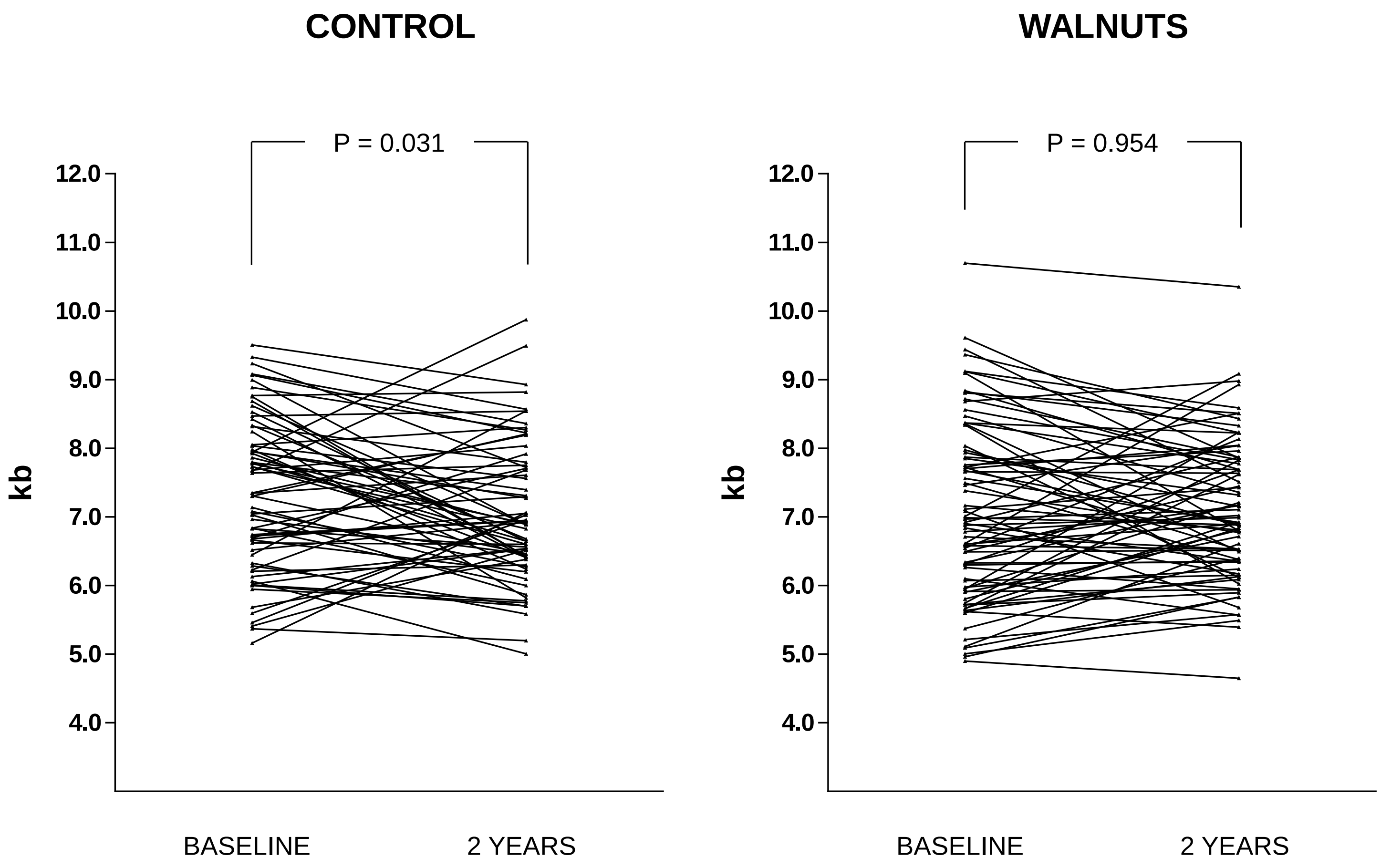

Comparisons between before and after the intervention (P obtained by repeated-measures ANCOVA with age, gender, and smoking in pack-years as covariates).

eTable 1. Baseline and 2 year red blood cell proportions of selected fatty acids by intervention group.

	Visit	Walnut group (n = 80)	Control group (n = 69)	P *
C14:0	Baseline	1.67 (1.35 to 1.99)	1.56 (1.21 to 1.91)	0.737
	2 year	1.78 (1.63 to 1.94)	1.76 (1.60 to 1.93)	
C16:0	Baseline	20.33 (20.11 to 20.55)	20.50 (20.27 to 20.74)	0.474
	2 year	20.34 (20.18 to 20.51)	20.65 (20.47 to 20.82)	
C16:1n-7 cis	Baseline	0.64 (0.59 to 0.68)	0.60 (0.55 to 0.64)	0.004
	2 year	0.52 (0.45 to 0.59)	0.63 (0.56 to 0.71)	
C18:0	Baseline	14.73 (14.38 to 15.08)	15.09 (14.72 to 15.47)	0.028
	2 year	13.87 (13.58 to 14.16)	13.49 (13.17 to 13.80)	
C18:1n-9 cis	Baseline	18.36 (17.99 to 18.73)	18.15 (17.75 to 18.55)	<0.001
	2 year	15.77 (15.50 to 16.04)	16.74 (16.45 to 17.03)	
C18:2n-6	Baseline	12.68 (12.31 to 13.05)	12.98 (12.58 to 13.38)	<0.001
	2 year	15.52 (15.13 to 15.92)	14.15 (13.72 to 14.57)	
C20:0	Baseline	0.22 (0.21 to 0.24)	0.24 (0.23 to 0.25)	0.073
	2 year	0.14 (0.14 to 0.15)	0.15 (0.14 to 0.15)	
C20:2n-6	Baseline	0.28 (0.26 to 0.29)	0.29 (0.27 to 0.30)	<0.001
	2 year	0.41 (0.38 to 0.45)	0.32 (0.28 to 0.36)	
C20:3n-6	Baseline	1.63 (1.57 to 1.70)	1.77 (1.70 to 1.84)	<0.001
	2 year	1.61 (1.54 to 1.69)	1.87 (1.79 to 1.95)	
C20:4n-6	Baseline	15.39 (15.06 to 15.72)	14.92 (14.56 to 15.28)	0.049
	2 year	16.11 (15.75 to 16.48)	16.14 (15.74 to 16.53)	
C20:5n-3	Baseline	0.92 (0.74 to 1.10)	0.79 (0.60 to 0.98)	0.682
	2 year	0.90 (0.83 to 0.98)	0.83 (0.75 to 0.91)	
C22:4n-6	Baseline	2.23 (2.13 to 2.33)	2.18 (2.07 to 2.29)	0.179
	2 year	2.37 (2.26 to 2.48)	2.39 (2.28 to 2.51)	
C22:5n-3	Baseline	1.60 (1.54 to 1.66)	1.61 (1.55 to 1.68)	<0.001
	2 year	2.01 (1.94 to 2.07)	1.68 (1.62 to 1.76)	
C22:6n-3	Baseline	5.07 (4.88 to 5.25)	5.02 (4.82 to 5.22)	<0.001
	2 year	5.01 (4.84 to 5.19)	5.43 (5.24 to 5.62)	
Ratio n-6 PUFA:n-3 PUFA	Baseline	4.41 (4.21 to 4.60)	4.45 (4.24 to 4.66)	0.281
	2 year	4.50 (4.32 to 4.67)	4.44 (4.25 to 4.63)	

Data are means (95% CIs). PUFA, polyunsaturated fatty acids.

* Interaction between time (baseline vs. retest) and intervention (control vs. walnut), obtained by two-way repeated measures ANOVA.
